# Social justice, epidemiology and health inequalities

**DOI:** 10.1007/s10654-017-0286-3

**Published:** 2017-08-03

**Authors:** Michael Marmot

**Affiliations:** Institute of Health Equity, Department of Epidemiology and Public Health, University College London, London, UK

**Keywords:** Equity, Health inequalities, Social determinants of health, Social gradient, Reverse causation

## Abstract

A lifetime spent studying how social determinants of health lead to health inequalities has clarified many issues. First is that social stratification is an appropriate topic of study for epidemiologists. To ignore it would be to ignore a major source of variation in health in society. Not only is the social gradient in health appropriate to study but we have made progress both in understanding its causes and what can be done to address them. Post-modern ‘critical theory’ raises questions about the social construction of science. Given the attack on science by politicians of bad faith, it is important to recognise that epidemiology and public health have a crucial role to play in providing evidence to improve health of society and reduce inequalities. Evidence gives grounds for optimism that progress can be made both in improving the health of the worst-off in society and narrowing health inequalities. Theoretical debates about ‘inequality of what’ have been helpful in clarifying theories that drive further gathering of evidence. While it is important to consider alternative explanations of the social gradient in health—principal among them reverse causation—evidence strongly supports social causation. Social action is by its nature political. It is, though, a vital function to provide the evidence that underpins action.

I was walking in the Mall in Washington DC. For Europeans, that is the area of DC that was sparsely populated during Donald Trump’s inauguration as US President—much to his chagrin and child-like attempts to lie about what was evident in plain view. In the section devoted to Martin Luther King Jr., I found this quote of his, King’s not Trump’s:

I believe that unarmed truth and unconditional love will have the final word in reality. This is why right, temporarily defeated, is stronger than evil triumphant.

As chair, I had been telling members of the Commission on Equity and Health Inequalities in the Americas, sponsored by the Pan American Health Organisation, that we needed ‘evidence-based policies presented in a spirit of social justice’. ‘Unarmed truth and unconditional love’, perhaps, is a more eloquent way of saying the same thing. Parenthetically, the Commissioners include distinguished lawyers, specialists in health diplomacy, health care administration, women’s health, the rights of indigenous people and a former US Surgeon-General, David Satcher. Apart from me, probably only one member of the Commission, would answer to the call: epidemiologist. What, you might ask, is an epidemiologist doing with such activities and in such company. You might ask, too, if the evidence in my ‘evidence-based policies’ would pass the epidemiology quality test.

An illustration from another scene: the Scottish Parliament. The context: the Health and Sport Committee of the Scottish Parliament taking evidence on health inequalities from experts—(then) Scottish Chief Medical Officer, Harry Burns, and me. One Scottish MP, having listened to my evidence, asked me:

‘What would *you* do if you were Chancellor of the Exchequer (UK Minister of Finance)?’

My response: ‘I know how lucky I am, but the people of Britain don’t know how lucky they are, that I am *not* the Chancellor. But what I would say to the Chancellor is that he should take no action that is likely to make health inequalities worse. Predictably, his changes to the tax and benefit system will increase the number of children growing up in poverty. Other things equal, that will have an adverse impact on health inequalities.’

I claim that I have the evidence to support such a statement but it bears little resemblance to what we teach students in epidemiology method classes. The argument runs like this. Evidence shows there is a gradient in the quality of early child development—the lower the socioeconomic position the worse do children perform on standard tests of cognitive, linguistic, social, emotional and behavioural development [1]. Much of this can be accounted for statistically by parenting activities [2]. The social gradient in early child development can be reduced by two strategies: reducing child poverty and by providing support for parents and families. Readiness for school predicts school performance [3]. Achieved educational level is strongly correlated with adult health and hence is a potent predictor of health inequalities. Such effects may be a direct consequence of education or may arise because education is associated with income, type of work, living conditions and psychological processes which are associated with health. Further, adverse child experiences are more frequent the lower the socio-economic position of parents. And adverse child experiences are linked with many predictors of adverse health outcomes: smoking, drug use, under-age sex and teenage pregnancy, domestic violence, mental illness and possibly physical illness.

What I do not have is a randomised controlled trial that shows that a reduction in child poverty now will reduce health inequalities when today’s children are older adults, sixty plus years from now. Such is not the nature of the evidence on health inequalities.

I have been concerned, nay obsessed, with social inequalities in health: how we understand the causes of the social gradient in health; and how action on the social determinants of health could improve population health and reduce avoidable health inequalities.

I have toiled happily in this vineyard for more than four decades. In the early years it was “pure” research. But yesterday’s pure research became today’s applied research and, increasingly, research aimed at understanding became enmeshed with efforts aimed at policy and practice. In this essay, I want to deal with issues with which I have wrestled along the way.

## Should “social class” be a proper concern of epidemiologists?

The answer for some is a clear no. Too vague and ill-defined, too freighted with political baggage, not an area for scientific enquiry. In this view, epidemiology, in investigating causation, should be about establishing exposure-disease relationships, not lamenting the ills of society. Poverty *is* lamentable and may be bad for health but let’s stick with more proximate causes that can be defined and measured. PM 2.5 can be measured, so can smoking and drinking and, with somewhat less precision, dietary intake; better still are biomarkers or SNPs. But poverty? It will mean something different in Zambia than it will in Glasgow; something different in 1950 from what it means in 2020. What does it mean, then, to be studying poverty and health? Or, when studying the social gradient in health, as I do, being in the middle of the socioeconomic hierarchy will have different implications in Kolkata than it will in Berlin.

Further, if the chain of reasoning that I laid out in the Introduction is the best I can do, it falls so far short of establishing causation, it gives the whole enterprise a bad name.

**Fig. 1:**
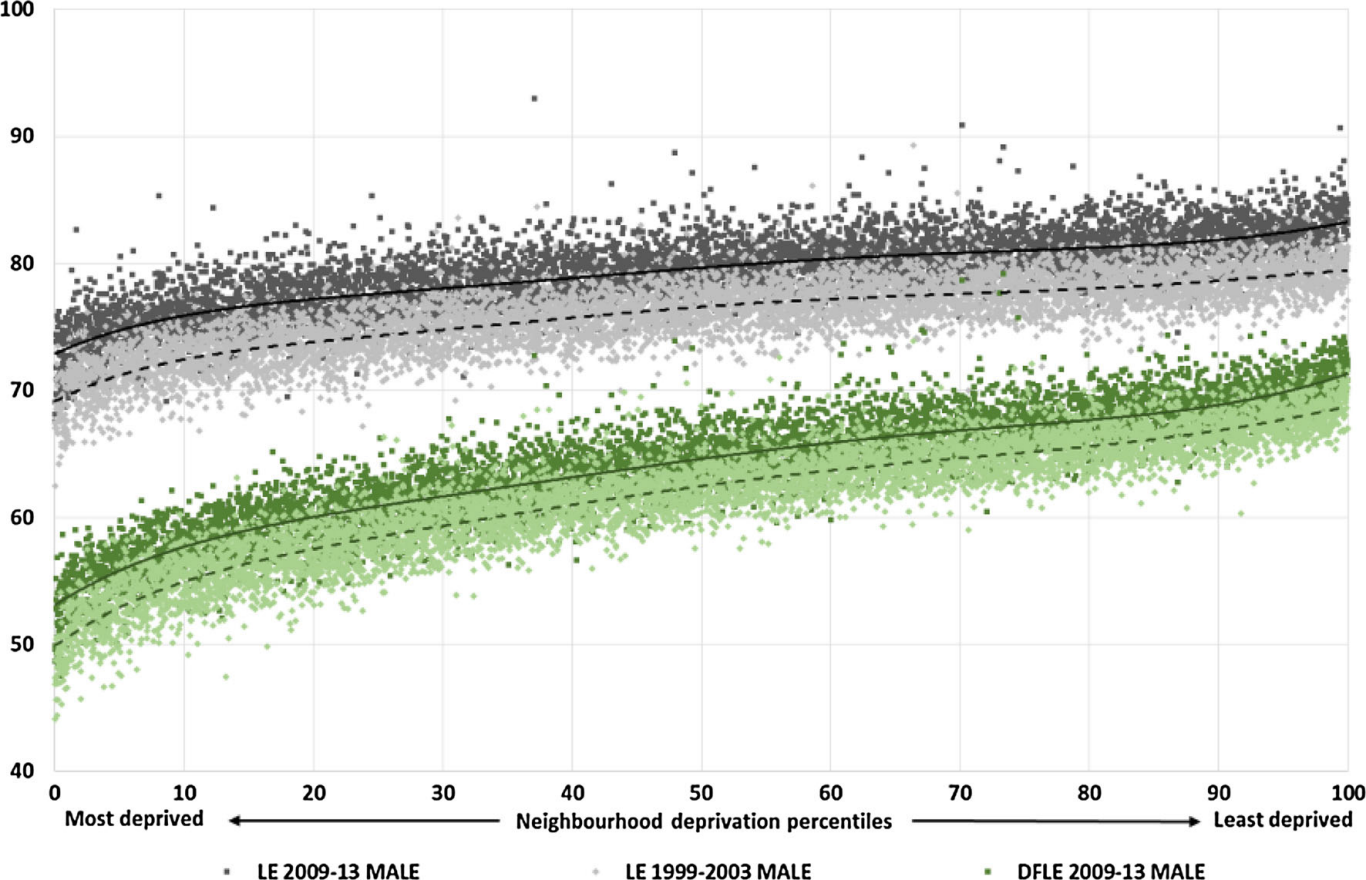
Life expectancy and disability-free life expectancy (DFLE) at birth, males by neighbourhood deprivation, England, 1999–2003 and 2009–2013

My response: look at the data. Figure 1 is an updated version of Fig. 1 from *Fair Society Healthy Lives*, the Marmot Review of Health Inequalities in England (1), that I reproduced at the beginning of my book, *The Health Gap* [4]. It plots life expectancy and disability-free life expectancy for neighbourhoods in England classified by neighbourhood deprivation. There is a remarkable social gradient: the more deprived the neighbourhood the worse the health. The gradient is steeper for healthy life than it is for expected length of life. Much of the scatter around the line is reduced if we produce a family of plots such as that in Fig. 1, one for each region of England. It is worse for your health to be socially disadvantaged in the North of England than it is in the South-East.

For the Marmot Review, we calculated that if everyone in England had the same death rates as the most advantaged, a total of between 1.3 and 2.5 million extra life years would be enjoyed by those dying prematurely each year. They would in addition have had a further 2.8 million years free of disability. Or, to put it slightly differently, if everyone in England had a mortality rate as low as those with university education, there would be 202,000 fewer deaths each year of people aged 30 and above.

We see gradients such as this in most countries for which data are available. And where the data are not available for adult health, we see gradients for under five mortality by household wealth [5].

To ignore the social gradient in health because you think that socioeconomic position, or degrees of deprivation, are vague round the edges, or because concern with health inequalities is politically motivated, is to ignore a major problem in health for all societies. Ah, might my critic respond, but is the link between education, or deprivation, and health causal, and, if so, what can we do about it. Answering those two questions is what I have been doing these last 40 years. The WHO Commission on Social Determinants of Health was, for me as chair, an important weigh station [6], and our European Review of Social Determinants and the Health Divide another [7]. Particularly with the surge in interest in many countries in the last few years [8] I have little doubt that we are making progress on both fronts—research and action.

Note that investigating health inequalities does not ignore research investigating whether exposure *a* is causally linked to health outcome *b.* In part, work on health inequalities depends on demonstration of such causes. I adopted Geoffrey Rose’s simple phrase, the *causes of the causes* [9]. Smoking is a cause of ill-health but why do smoking and other unhealthy behaviours follow the social gradient—what is the cause of the cause. Exposure to high levels of PM2.5 is a cause of ill-health, but why are people lower in the social hierarchy more likely to be exposed—the cause of the cause.

If looking for causal intermediaries between position on the hierarchy and ill-health takes us downstream, then looking for the causes of the causes needs to take us upstream into the nature of society that leads to, and tolerates, stark inequalities in conditions of daily life; and inequities in power, money and resources that give rise to these inequalities in conditions in which people are born, grow, live, work and age—language that we used in the report of the Commission on Social Determinants of Health.

A further word on language is necessary. For good or ill, those of us concerned with social inequalities in health have stopped using the term “social class”. It has baggage and multiple meanings. Do we mean Marx’s two great classes, bourgeoisie and proletariat—where ownership, or not, of the means of production is key? Or perhaps the linked Weberian concept of a category of men who have in common a component of their life chances? Or an Erikson-Goldthorpe notion of span of control at work? People using the term “social class” might mean or all or some of these. It is unclear.

We’ve also stopped using the term SES, socioeconomic status, because it implies that differences in health between social groups can all be attributed to differences in status. Full disclosure, I contributed a little to the confusion by writing a book with the title, *Status Syndrome* [10] which actually said little about ‘status’ and health. It said much more about how lower social position was linked to low control over life and less opportunity for social participation than it did about perceived status. That said, there is evidence that perceived status may be important [11].

More common now is to use the neutral-sounding term, socioeconomic position. It makes no judgement as to the theoretical basis of the classification—a mixed blessing. No doubt, in time, it too will take on baggage. But for the moment it serves to classify individuals along a social gradient and allows investigation both of how they got there, and the health consequences of being there: causes of the causes of the causes.

## Positivist?

As always, discussions about language are really discussions about ideas. When I first started researching socioeconomic differences in health, two common terms of abuse from social scientists were ‘positivist’, and ‘atheoretical’. If positivism means focussing purely on empirical observations and ignoring the ideology and structures of knowledge that we bring to our studies then such critique surely has merit. But beware. If so-called ‘critical theory’ leads to a post-modern questioning of the very possibility of objective truth, then in an age of Donald Trump where there are facts and ‘alternative facts’, where ‘truth’ is whatever feels convenient and best serves political advantage, where global warming is a hoax perpetrated by the Chinese, we are in grave danger [12]. For example, destitution is bad for health. Post-modern critical theorists may call both ‘destitution’ and ‘health’ social constructions, and question the methods we use to establish a link between the two. But if men in a deprived part of London have life expectancy 18 years shorter than men in a rich part, that is a fact that should claim our attention, stimulate research, and inspire calls for action.

To put it more plainly, scholars and political activists with good motivation—critiquing the very nature of our knowledge, and showing how science is part of power structures—play into the hands of political charlatans, whose motivation is much more sinister. When Trump says that the murder rate in the US is at an all-time high, he is not being post-modern, he is lying. The murder rate is near to an all-time low. He is lying to make a political point—in his inaugural address he spoke of ‘carnage’. During the 2016 US election campaign, when official figures said that unemployment under the last year of the Obama Presidency was around 5%, Trump said it was fake and claimed that the real figure was five, six, eight times that. When Trump was President, and the unemployment was still at around 5% he took credit for reducing it. His spokesman quoted the President as saying, the figures were fake then; they are real now. Hahaha! That laugh sent shivers up and down many spines. Golly, how funny that you can mess around with official figures at will. The planet warming? Nah. A conspiracy of left-wing scientists.

Facts matter.

## Atheoretical?

As for being ‘atheoretical’, initially I was somewhat defiant. I said I didn’t care much whether the social gradient was predicted by education, deprivation of the area or, in the case of my Whitehall studies, grade of employment [13, 14]. The fact was that each of them showed health to follow a social gradient. The question was why.

The theories that I find useful now perhaps are not very grand or high level, but they are indispensable. First, Max Weber thought of social stratification not so much as a characteristic of a person, their status for example, but a characteristic of society. We can measure individuals’ social position but that should not blind us to the fact that societies differ in their degrees of inequality.

Second, and related, in the Commission on Social Determinants of Health we explicitly drew attention to what we called the structural drivers of health inequalities: inequities in power, money and resources. These are features of societies not simply of individuals within those societies. It draws heavily on Weber. Drawing on an old, but still fresh text of Mervyn Susser [15], we need to get the level of analysis right. We measure individual socioeconomic position and examine the extent to which its link with health can be ‘explained’ statistically by smoking, or other risk factors. But that won’t tell us whether democracy, respect for human rights and socially inclusive societies are good for health. We need, at least conceptually, a different level of causal thinking for that.

Third, I have spent decades wondering which is more important: relative or absolute poverty. I am not alone. Peter Townsend and Amartya Sen tussled with this and each other three decades ago [16, 17]. In a low-income country, there is little difficulty in seeing how absolute poverty damages health. Even in a high-income country, if people low in the hierarchy have insufficient money to buy food and pay rent, absolute poverty must be important. But the social gradient surely implies relative poverty. Even here it could be questioned. The gradient shown in Fig. 1 could arise if richer areas simply had fewer people in absolute poverty. That could not, however, readily account for the gradient in health by level of education. The latter implies relative disadvantage.

Amartya Sen solved it by arguing that relative deprivation with respect to income corresponded to absolute deprivation with respect to capabilities [18]. My way of putting this is that it is not so much what you have that is important for health, but what you can do with what you have [4]. A family in a low income area of Baltimore Maryland has a median household income of $17,000. By US standards, this is poverty. Yet, on a global scale they are fantastically rich. Gross National Income per capita, adjusting for purchasing power, in Costa Rica is $14,000. Life expectancy for men in Costa Rica is 77; in the poor part of Baltimore 62. The poor of Baltimore may be rich compared to the Costa Rican average but they are poor relative to the US and that translates into absolute disadvantage. In the poor part of Baltimore life chances are sorely diminished: single parent families, low levels of education, high probability of arrest by the age of 17, high probability of being shot. It is not so much what they have but what they can do with what they have; and that will depend on the nature of society.

Sen’s ‘capabilities’ are linked to his basic notion of freedom to lead a life one has reason to value [19]. Before I knew of Amartya Sen, building on the work of Karasek and Theorell [20], I had shown in the Whitehall II study that low control at work provided a partial explanation of the social gradient in health [21]. Sen’s freedom and capabilities gave me the impetus to generalise. In the Commission on Social Determinants of Health we put empowerment at the heart of what we were trying to achieve: material, psychosocial and political.

**Fig. 2:**
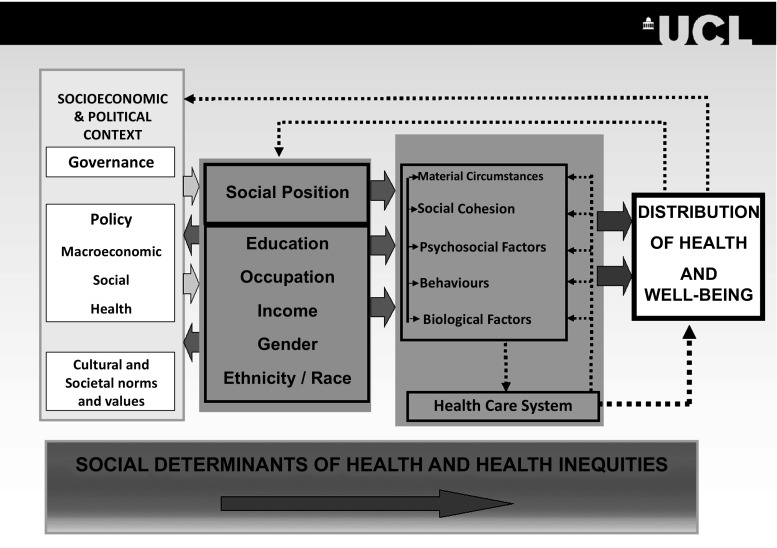
Model of causation of social determinants of health. *Source*: CSDH Final Report, WHO 2008, adapted from Solar and Irwin (Unpublished report WHO Geneva)

If theory is a way of organising one’s thoughts, an explanation of how the world works, and thus an aid to understanding and a guide to action, then the charge of being atheoretical is one to take seriously. If not a theory, then at least a model. Figure 2 was developed for the Commission on Social Determinants of Health [22]. It illustrates the multi-level nature of our thinking. Assembling the evidence to support this model is, of course, a major challenge.

Parenthetically, I have never described myself as a ‘social epidemiologist’, although others might. I am concerned at the health of populations and inequalities in health. The evidence takes me in the direction of looking at how society impacts on health. That said, I feel a sense of kinship and shared orientation with those who have carved out the discipline of social epidemiology [23].

## But why do you need to look at inequalities? Take action on the causes and everyone’s health improves

This argument, too, has merit. Dirty water leads to preventable illness. Clean up the water supply and everyone benefits. You don’t need to lament that poor people were less likely to have access to improved water supply, and that this contributed to inequality. Simply do it for everybody.

Similarly with non-communicable diseases. If smoking rates decline everyone benefits. There may even be a reduction in inequality because smoking-related diseases follow the social gradient, more common lower down.

Look again at Fig. 1, however. It plots the gradient for the years 1999–2003, and again, ten years later, 2009–2013. Health has improved for everyone over the decade, a welcome societal achievement. When I protest, but what about inequalities, the response has been: What are you? Some kind of naïve egalitarian? Go home, lie down and get over it.

As Fig. 1 shows, at the same time health improved for everyone, the gradient did not change. We need to have two societal goals: improve health for everyone and reduce inequalities.

One way of thinking about this is the trade-off between efficiency and equity. For example, when Manolis Kogevinas worked with me years ago, he showed that for many cancer sites, people of low socioeconomic position had worse survival than those of high position [24]. One possibility is that the poor have less favourable response to treatment. There are other explanations—later presentation, worse access to treatment, less compliance—but let’s stay with biological differences for the moment. For efficiency, greatest health gain for a given quantum of effort and expenditure, focus on the more advantaged group. Forget the poor. Equity, of course, directs one in a different direction. But then, it is crucial to make sure that interventions achieve the desired health improvements. If the intervention is ineffective, equity is little served. The challenge is not to withhold benefits from the rich, but to reduce inequalities at the same time as benefitting the whole population.

## The poor are always with us

In all societies there will be the poor, relatively and absolutely. More accurately, all societies have social and economic inequalities. If the health gradient arises because of these inequalities won’t there always be health inequities? In which case shouldn’t we stick with the one goal, improving health for everybody, and forget the second one of reducing inequalities.

I have two answers. First, look again at the two curves showing the gradient in life expectancy in Fig. 1, and focus not on the extremes but on people towards the top and those below the middle. In the earlier period, 1999–2003, life expectancy of men in affluent areas, at the 80th centile, was around 78, and for men in more deprived areas, the 30th centile, was 74. Ten years later life expectancy in the somewhat deprived areas at the 30th centile had increased to 78. The health of the poorly off in 2010 is as good as that of the well-off ten years earlier.

In 2000 if, contemplating the social gradient in health, we had said that we could get the health of people near the bottom of the social gradient up to that of people near the top, it would be hailed as a major boon to the disadvantaged members of society. It was done. It just took ten years. The hitch, of course, was that in that time health for those near the top had improved, too. The lesson I take from this is that if the health of the poor can be improved quickly, then there is nothing fixed about inequalities in health. The fact that the slope of the health gradient did not change despite overall improvements in health suggests we need to look upstream to social determinants of health inequity.

My second answer to “the poor are always with us” argument is that all societies do have social gradients in health but the slope varies. In the context of a European Review of health inequalities [25], we looked at life expectancy at age 25 by education in 15 different countries [26]. The countries of Central and Eastern Europe had low average life expectancy and big inequalities. Sweden, Norway, and Mediterranean countries had long average life expectancy and smaller inequalities. We need to move from an Estonian and Hungarian level of health inequity to a Nordic or Mediterranean level.

In the English Review we coined the term proportionate universalism. We were, I am, convinced by the evidence that one of the secrets to good health in Nordic countries is a commitment to universalism [27]. In Anglo-Saxon countries the default position in social policy is to focus on the worst-off. Proportionate universalism is our effort at combining these two approaches. We want universalist policies that include everyone, but effort has to be proportionate to need.

## Inequality of what?

A much-used measure of income inequality in economics is the Gini coefficient. It measures how far the income distribution of individuals strays from the line of equality: 1% of the population having 1% of the total income; 20% of the population having 20%; 90% having 90%. Inequality, here, applies to individuals. It turns out that when economists use the term ‘inequality’ they do mean the variability among individuals, the total variance. When we refer to inequality in public health, certainly in the UK, we mean variability among social groups. The first thing, then, is to get the language straight so that we can communicate.

But, as above, a linguistic issue is commonly an ideological issue. When we published the report of the Commission on Social Determinants of Health (CSDH) in 2008, I was asked to make a presentation about it at the London School of Economics (LSE)—one of the jewels in the crown of British Universities. A distinguished economist, invited to comment on the report, observed that for every mention of individual differences in health, there were *n* mentions of social inequalities, where *n* was a large number. Well, of course, the name was on the cover. The causes of individual differences in health may be different from the causes of group differences. There may be overlap, but differences, too.

In the CSDH, we adopted the WHO usage that flows from Dahlgren and Whitehead [28], and used ‘health equity’ to refer to those systematic inequalities in health between social groups that are judged to be avoidable by reasonable means. If not avoided they are inequitable. Hence our phrase that graced the cover of the CSDH Report: “Social injustice is killing on a grand scale”.

My own particular concern has been the social gradient in health, defined by socioeconomic position, and the importance of social determinants in leading to these health inequities. A persistent question is what about racial/ethnic differences in health. My starting position is that the same set of social determinants are likely to account for the differences in health between indigenous and non-indigenous people in Australia, New Zealand, Canada, the US and elsewhere. Racism, discrimination and stigma will be causes of unequal distributions of these social determinants both between and within ethnic groups.

There is, of course, another crucial question when it comes to ‘inequality of what?’ which I will touch on briefly: equality of opportunity or equality of outcome [29]. If politicians or social commentators are going to endorse any principle of equality, equality of opportunity is the easiest. It appeals to natural justice. In theory, every mother’s son can be US President—harder for a daughter. In practice, the conditions in which people are born and grow are so dramatically different that equality of opportunity is a chimera. That said, politicians love to endorse equality of opportunity to establish their commitment to fairness.

Health, though, is an outcome. As Fig. 2 shows it is the outcome of a chain of social processes. Gaining commitment to equality of outcomes is a different, and much more difficult proposition. It will require a focus on outcomes along the causal chain. For example, making sure there are school places for all is a step towards equality of opportunity. But we should not stop there. We need to look at outcomes. In all countries, there are social gradients in performance on standard tests. They are shallow in Finland, steep in the US. As a social goal, with health equity in view, we should be seeking to reduce the social gradient in school performance.

## The mind is an important gateway by which the social environment influences health and health inequalities

In the beginning were Farr, Stevenson and Florence Nightingale—statisticians who pioneered the study of social determinants of health and health inequalities in England. They begat Black [30]. Black begat Acheson [31]. Acheson begat Marmot [1]. I am, of course, referring to successive reports, commissioned by governments, on health inequalities in England. In between was Whitehead [32]. Her report was commissioned by the Health Education Authority, but a Conservative government wanted to refuse publication, and Penguin happily stepped in.

As a member of the Acheson Inquiry, I pushed strongly that we should consider not just poverty and health, but the gradient. Sir Donald Acheson said: but if we consider the gradient, we will have to consider psychosocial influences on health. Correct. And we did.

In my Marmot Review, psychosocial influences ran all the way through our six domains of recommendations: early child development, education, employment and working conditions, having enough money to live on, healthy environments in which to live and work, a social determinants approach to prevention.

Briefly, there had been a vogue to contrast neo-material influences on health with psychosocial influences and belittle the latter [33]. I was never convinced [34]. Take, for example, the fourth recommendation from *Fair Society Healthy Lives* [1], everyone should have the minimum income necessary for a health life. If one were contrasting material or neo-material influences with psychosocial, having insufficient money sounds rather “material”. But why does it damage health? Living in a cold home, for example, is bad for respiratory health; it also has an adverse effect on children’s mental health and school performance. Parents in poverty are less likely to engage in nurturing activities with their children. Mental illness and alcohol problems are more frequent with poverty. Why would you want to make such a sharp distinction between material and psychosocial influences? There is now much interesting work on the psychology of poverty [35, 36].

In fact, psychosocial influences are important in social determinants of health and health equity in at least four ways. First, early child development and education set the context for what happens through the rest of the life course; they influence opportunities and choices, work and social relationships. What are these if not processes in the mind. Second, behaviours influence health: drug use, alcohol, smoking, diet, exercises. Third, stress pathways to physical disease are crucial. Fourth, mental illness is an important consequence of social disadvantage.

A paper by Case and Deaton drew attention to the rise in mortality in non-Hispanic whites, aged 45–54 in the USA—the fewer the years of education the steeper the rise [37]. The causes of death constituting this rise were: poisonings due to drugs and alcohol, suicide and alcoholic liver disease—all psychosocial. I would not stop there, but ask what are the structural (neo-material) causes of this epidemic of disempowerment.

## Causal arrows

Which way do the causal arrows go: from wealth to health, or from health to wealth? I am slow to anger and with the self-delusion that I am considerate of the opinions of others. But my tolerance has been exercised by this debate. At times, I have gone overboard and said: ALL the evidence points to social conditions causing ill-health not ill-health causing social conditions. That has to be an overstatement. If ill-health stops someone working and family income falls, the arrow is running from health to wealth. But we should not stop there and ignore the evidence of social causation, of the arrow running from wealth to health.

I once asked a senior economist, President of a University, why it is that, as a group, economists have as a starting point that the causal arrow goes from health to wealth. The President said: because the equations are easier to solve if you put income on the left-had side of the equation and health on the right-hand side. Really?

Certainly, an economist that I collaborate with happily told me that one of the effects of our collaboration is that his students who formerly were taught that health is an input to income and wealth are now taught that the arrow can run both ways. I suppose that even a modest achievement is an achievement.

The implications of these views of the direction of the arrows for policy are profound. The Journal *Social Science and Medicine* commissioned 8 groups of authors to write commentaries on the Marmot Review, *Fair Society Healthy Lives* [1]. My colleagues and I were invited to respond to these 8 commentaries. What follows, in this section, is from our response in *Social Science and Medicine* [38].

Six of the commentaries are in little doubt that we have enough evidence to take action on social determinants of health; although all, like us, want a stronger evidence base. The other two commentaries thought we had the model wrong [39, 40]. Their starting position, like that of many economists involved in the social determinants debate, is that peoples’ health determines what happens to them. The Review’s starting position was that what happens to people has a cumulative effect throughout their life course, progressively affecting their health.

At the time, I had been reading Dickens’s, *Hard Times.* I took a page on housing:

In the hardest working part of Coketown,… where Nature was as strongly bricked out as killing airs and gases were bricked in… where the chimneys, for want of air to make a draft, were built in an immense variety of stunted and crooked shapes (pp. 65–66).

And then a description of working conditions in a northern mill town:

all the melancholy-mad elephants, polished and oiled up for the day’s monotony, were at their heavy exercise again…. Every man was in the forest of looms where Stephen worked to the crashing, smashing, tearing piece of mechanism at which he laboured.

Should we really assume, that these dark satanic mills and airless places, rather than causing terrible illness and shortened lives, selectively employed sick people and those whose backgrounds accounted for all their subsequent illness? That subsequent improvement in living and working conditions, thus abating Victorian squalor, and associated improvements in health were correlation not causation? That while medical care improved health, housing also got better, and the public health profession mistook the improvement in housing and working conditions for causes of improved health?

If proponents of this set of assumptions dropped their guard for a moment and accepted the evidence that air pollution, crowded living conditions, ghastly working conditions were causes of ill-health in Victorian times why, a priori, do they start from the position that living and working conditions are not a cause of ill-health in the twenty-first century? Why do they appear to assume that Fig. 1 in the Review, reproduced above, linking neighbourhood deprivation to disability-free life expectancy could all be due to a remarkable ability of people to choose places to live depending on their level of health—ill health leads to neighbourhood income, in other words? Which of their many coefficients proves that? At a regional level, it is equally difficult to see how selection explains why the social gradient is widest in the North East and narrowest in the South West, as both regions have a history of out-migration of those needing to find employment.

This disagreement between commentators is not just about evidence. It is also about ideology. We think that the health gradient in Fig. 1 is a powerful demonstration of the graded relation between social and economic conditions and health. We are chastised, by Canning and Bower, for wanting a fairer society to put it right. Instead, they offer the following:

The health gradient should be seen as a flashing alarm that our health systems are failing to deliver cost effective health care and a call to allocate health sector resources more effectively.

Why should it? Where is the evidence for their counter assertion? They are not being more rigorous about causation than we are, as they claim. They simply have a different starting position. This is ideology dressed up, condescendingly, as methodological rigour. We would go further. Given the vast research resources that have gone into evaluating medical interventions, the lack of clear evidence that the main cause of the social gradient in health is differential access to health care, may mean that, indeed, it is not lack of health care that is the cause of the problem.

## Not political enough…

The economists’ criticisms seem to be that, in part, our science is clouded by political motivation. A different kind of criticism is that I have not been political enough; that indeed politics can be studied as a determinant of social determinants of health. When we published the report of the CSDH with “Social Injustice is Killing on a Grand Scale” on the cover, Vicente Navarro praised the report [41]. He then went on to chide us for not going far enough. His view was that we know who the killers are. My response was that we had reviewed the evidence on social determinants of health. I was perfectly content if others wanted to take it to a more overtly political level. We went so far as to say that health inequalities resulted from a toxic combination of poor quality social programmes, unfair economic arrangements and poor governance—and gave the evidence to support those contentions.

A related challenge to us was: “Isn’t the problem really capitalism”. My response to this, too, is likely to disappoint. I point out that the countries with the best health, longest life expectancy, are Japan, Iceland, Sweden, and now Hong Kong—all capitalist countries. Evidence would suggest that it is not so much capitalism, *per se*, that is the problem but how particular capitalist societies are operated. Runaway inequalities may be a feature of the US and UK; much less so, Germany and France, let alone the Nordic countries.

## … but social enough

I have spent the majority of my academic life as a researcher, much of it on the social determinants of health. But something happened. I started wagging my finger. With growth in the quantity and quality of evidence on social determinants of health, I became more strongly of the view that failure to take action on avoidable health inequalities was unjust. If we know what to do, and we don’t do it, society is at fault. Hence my wandering in the Mall and being entranced by quotes from Martin Luther King Jr. I know the argument. The more we become committed to a position the less objective becomes our science.

I was once asked by a BBC Radio Interviewer: Why should I believe you? Perhaps you are cherry-picking the evidence to support your view point.

It was an astute question. I responded that, as with every scientist, I was committed to my theories, hypotheses and evidence. But the nature of science is that if we are wrong, we are shown to be wrong. If a scientist doesn’t modify his views in response to counter-evidence he becomes irrelevant. Sometimes it may take a while, but it happens.

I began this essay in the Washington Mall with Martin Luther King. On the way, I indulged in a diatribe about the importance of facts. When we published my English Review, *Fair Society Healthy Lives,* in my note from the chair, I referred to the fact that the CSDH had been criticised by one country representative as ‘ideology with evidence’. I said that we do have an ideology. Health inequalities that are avoidable and are not avoided are unjust. Putting them right is a matter of social justice. But the evidence really matters.

Evidence-based policies presented in a spirit of social justice.
